# Effects of Encapsulated Propolis on Blood Glycemic Control, Lipid Metabolism, and Insulin Resistance in Type 2 Diabetes Mellitus Rats

**DOI:** 10.1155/2012/981896

**Published:** 2011-06-12

**Authors:** Yajing Li, Minli Chen, Hongzhuan Xuan, Fuliang Hu

**Affiliations:** ^1^College of Animal Science, Zhejiang University, No. 268 Kaixuan Road, Hangzhou 310029, China; ^2^Department of Applied Engineering, Zhejiang Economic and Trade Polytechnic, Hangzhou 310018, China; ^3^Laboratory Animal Research Center, Zhejiang Traditional Chinese Medicine University, Hangzhou 310053, China; ^4^School of Life Science, Liaocheng University, Liaocheng 252059, China

## Abstract

The present study investigates the encapsulated propolis on blood glycemic control, lipid metabolism, and insulin resistance in type 2 diabetes mellitus (T2DM) rats. The animal characteristics and biological assays of body weight, fasting blood glucose (FBG), fasting serum insulin (FINS), insulin act index (IAI), triglycerides (TG), total cholesterol (TC), high-density lipoprotein cholesterol (HDL-C), and low-density lipoprotein cholesterol (LDL-C) were measured and euglycemic hyperinsulinemic glucose clamp technique were used to determine these effects. Our findings show that oral administration of encapsulated propolis can significantly inhibit the increasing of FBG and TG in T2DM rats and can improve IAI and M value in euglycemic hyperinsulinemic clamp experiment. There was no significant effects on body weight, TC, HDL-C, and LDL-C in T2DM rats treated with encapsulated propolis. In conclusion, the results indicate that encapsulated propolis can control blood glucose, modulate lipid metabolism, and improve the insulin sensitivity in T2DM rats.

## 1. Introduction

Type 2 diabetes mellitus (T2DM) is a metabolic disease characterized by an elevated blood glucose concentration that results from inadequate insulin action in insulin-sensitive tissues and from abnormal insulin secretion. The number of people diagnosed with T2DM is increasing at an alarming rate. In the USA, Europe, and most westernized countries, T2DM affects ~6% of the population [1]. In big cities of China, the attack rate also reaches 6% [2]. According to a widely accepted estimation, the number of diabetic patients (most of them with T2DM) can reach 366 million by the year 2030, promoted by a dramatic increase in the incidence of obesity and a sedentary lifestyle [3]. T2DM, especially its developing complications, carries a very poor prognosis and may lead to a dramatic decrease of life expectancy. Economical analyses of late complications made it clear the total cost of care of patients with T2DM and its complications is very high. Thus, much interest has been focused on the development of alternative medicinal foods, which includes screening of natural bioactive compounds with the ability to improve glucose control and lower the risk of complications [4, 5].

Propolis is a safe natural products which is a resinous hive material collected by honeybees from various plant sources. It is a popular folk medicine possessing a broad spectrum of biological activities, such as anticancer, antioxidant, antiinflammatory, antibiotic, antifungal, and antihepatotoxic [6–10]. In recent years, there have been many surveys carried out that propolis has hypoglycemic activity [11–13] and has some positive effects on diabetic complications [14, 15]. Researchers explored the way of propolis resist oxidative damage [16], enhance body immunity, and influence the metabolic enzyme to reveal its therapeutic effects on diabetes mellitus [17]. However, the effects of propolis on glycometabolism, lipid metabolism, and insulin resistance in T2DM rats are uncertain. 

Our previous studies showed that propolis ethanol and water extracts exert a positive effect in diabetic animals [18–20]. However, as propolis is a strongly adhesive, resinous mixture of insoluble or slightly soluble substances with bitter taste, the application of propolis as a health-promoting agent has been limited [21]. To improve the solubility and bioavailability of propolis, an encapsulation techniane has been used [22]. In this study, we explored the effects of encapsulated propolis on the metabolic and insulin-resistance state of type 2 diabetic rats. The aims were to, (i) verify the positive effects of encapsulated propolis on modulate glucose and lipid metabolism in T2DM rats, (ii) investigate its effects on insulin resistance, and (iii) investigate the optimal concentrations and intake period for T2DM treatment.

## 2. Materials and Methods

### 2.1. Laboratory Animals

Male Sprague-Dawley rats of about 270~370 g were provided by the Shanghai Laboratory Animal Center of the Chinese Academy of Sciences (Certificate of animal quality: Zhong Ke Dong Guan No. 003). All animals were housed in a rat breeding room with air conditioning and an air defecation filtering device. The rats were allowed a standard pellet diet, free access to tip filter water and maintained at 23 ± 2°C, 50–70% humidity, a noise level <50 dB, and with 12 h dark/12 h light cycles. All the conditions were keeping with the Helsinki accord and the experimental protocol was approved by the Animal Ethics Committee of Zhejiang University.

### 2.2. Drugs and Reagents

Propolis was obtained from colonies of honeybees, *A. mellifera L.*, in north China in 2009, and the main plant origin was poplar (*Populus* sp.). Encapsulated propolis was prepared according to a published technical [22] with minor modifications: raw propolis was pulverized in a mill then extracted with 80% ethanol to prepare a 1% ethanol extracts of propolis(EEP), 1% EEP was stirred into 5% beta-cyclodextrin aqueous solution, and the mixed liquor was sonicated for 15 min at 25 kHz; after being sonicated, the sample was rotary evaporated to remove the ethanol, and spray-drying technique was used to obtain the encapsulated propolis. Encapsulated propolis used in this study possesses a grain size about 210 nm with 98.82% encapsulation rate and 29.65% drug-loading rate of EEP and showed a strong dissolubility in water. The total flavonoids were used as the marker for quality control of encapsulated propolis, and the content of it was 93.20 mg/g [23]. Streptozotocin (STZ) was obtained from Alexis Corporation; pioglitazone HCL tablet was obtained from Hangzhou Huadong Medicine Group Co., Ltd.; heparin Sodium Injection and Insulin Injection were obtained from Nanjing Xinbai Pharmaceutical Co., Ltd.; blood glucose test kit, TC test kit, TG test kit, and HDL-C test kit were obtained from Shanghai Shenergy Biotechnology Co., Ltd.; LDL-C test kit was obtained from Shanghai Fosun Long March Medical Science Co., Ltd.; insulin radio-immunity test kit was obtained from Beijing Kemei Dongya biotechnology Co., Ltd.

### 2.3. Induction of Type 2 Diabetic Mellitus (T2DM) Rat Model

During experimental session, male Sprague-Dawley rats (*n* = 100) were fed with a high-fat diet (HFD) consisting (as a percentage of total kcal) of 41% fat, 41% carbohydrate, and 18% protein. And injected with low-dose STZ 4 times every 6 days to induce the animal emerge T2DM symptom [24]. Briefly, all animals were fasted over night and then injected with STZ into the tail vein via a temporary indwelling 24-gauge catheter. The doses of STZ for each injection were 10 mg/kg, 5 mg/kg, 20 mg/kg, and 10 mg/kg, respectively. Animals had free access to food and water during each interval of STZ injection. After a week of the last STZ injection, a casual blood glucose, 12 h-fast glucose, water intake, and urine output were measured. The animals showing a casual blood glucose levels >11.1 mmol/L and with the symptom of polyuria and polydipsia were considered diabetic (*n* = 68, and 60 within them were selected for next studies).

### 2.4. Grouping and Drug Administration

T2DM rats were randomly divided into 5 groups via fasting glucose and fasting body weight (*n* = 12): model control group, low-, middle- and high-dose encapsulated propolis group, and positive control group. These 5 groups were continued on their HFD for the duration of the study. Select another 12 normal male SD rats as normal control group and fed with a normal chow diet (consisting of 12% fat, 60% carbohydrate, and 28% protein). Low, middle and high dose encapsulated propolis group were administrated with encapsulated propolis 50, 100, 200 mg/kg·d, positive control group were administrated with an insulin sensitizer, pioglitazone HCL tablet, 10 mg/kg.d, normal and model control group were administrated with filtered water at the rate of 1 mL/100 g.d. The administration continued for 10 weeks.

### 2.5. Biological Assays

The animal's fasting body weight was observed once a week. Every 2 weeks, blood was collected from rats' vena orbitalis posterior, and the serum was centrifuged at 3000 rpm to test FBG and TG. At the end of the study, FINS were measured and insulin action index (IAI = 1/FBG×FINS) were calculated [25], the values were transformed by taking the natural logarithm (Ln), TC, and HDL-C and LDL-C, were also determined.

### 2.6. Euglycemic Hyperinsulinemic Glucose Clamp Technique

Euglycemic hyperinsulinemic glucose clamp technique is a highly sophisticated labor-intensive method for the determination of in vivo insulin sensitivity; we performed glucose clamp studies in subgroups of rats. 12 T2DM rats (6 treated with EP and 6 as model control) and 6 normal rats were surgically prepared for the clamp procedure and then gavaged with encapsulated propolis 100 mg/kg·d or with filtered water 1 mL/100 g·d. for 6 days. All animals underwent a fasting period of 12 h and were anaesthetized with an intraperitoneal injection of sodium pentobarbital 65 mg/kg. All rats were placed on their back, then the left common carotid artery and the left jugular vein was exposed, a polyethylene tube were cannulated for collecting blood samples and administration of insulin and glucose. The technique used for the glucose clamp was the same as that described previously [26]. Briefly, each unconscious rat was placed in a foam plastic jacket that allowed movement of all four limbs and forward vision during the euglycemic clamp. Insulin infusion was commenced using an infusion pump (50 mU/kg at the rate of 0.1 mL/min) so as to maintain blood glucose at the basal level. After 1 min of insulin infusion, the first test glucose sample was determined and glucose infusion was commenced at the rate of 5 mg/kg/min. The infusion lasted for 120 min. Ten microliters of arterial blood glucose was sampled at 5 min intervals for determination of the blood glucose level. The average rate of glucose infusion (GIR) for the last 35 min was taken as the index of insulin sensitivity (M value) of each rat.

### 2.7. Data Analysis

Data were reported as means ± standard deviations. Prior to analysis, Levene's test and the Kolmogorov Smirnov test were used to test for homogeneity of the variances and normality of the response variables, respectively. All the response variables passed tests of normality (Kolmogorov Smirnov test: *P* > .10). Since homogeneity of the variances was not meet for all the variables (Levene test: *P* < .01), we used the *ln* transformation to stabilize the variances. The *ln* transformed response variables were used in the statistical analyses. Comparisons between groups over six time periods were made using time repeated measures ANOVA with the six time periods as the within-subjects factor and groups as the between-subjects factor. Significant effects were further examined using Tukey's multiple pairwise comparisons tests with a probability level of *P* < .05 being considered as significant. MANOVA procedures using Wilk's lambda were used to test for overall differences in response variables between the time periods and for interaction effects between time periods and groups.

## 3. Results

### 3.1. Animal Characteristics

**Figure 1:**
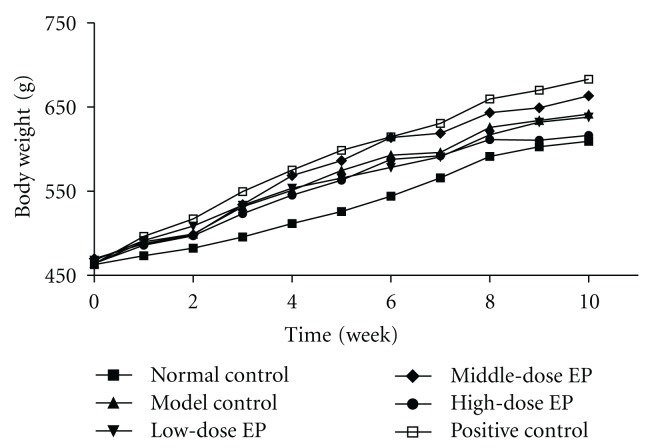
Effect of encapsulated propolis treatment on body weight in T2DM Rats. Values are expressed as the means (standard deviations not shown).

Figure 1 shows the body weight of normal rats and T2DM rats treated with or without encapsulated propolis or positive drug. The results show that there were no differences in body weight at the beginning of drug administration (*P* < .05), at the following 10 weeks period of drug administration, the body weight of each group increased gradually. Three weeks after drug administration, the body weight of model control group increased significantly than normal control group contemporary (*P* < .05, *P* < .01), and body weight of encapsulated propolis treated groups increased at equal pace with model control group (*P* > .05); however, the body weight of T2DM rats treated with positive drug increased quicker than model control group, and had significant difference on 7th and 10th week of drug administration (*P* < .05).

### 3.2. FBG in T2DM Rats Treated with Encapsulated Propolis

The means and standard deviations of encapsulated propolis over six time periods on FBG in T2DM rats are given in Table 1. The results of the time repeated measures ANOVA and MANOVA show significant group effect on fasting blood glucose (*F* = 39.4, *df* = 5,66, *P* < .0001), significant time effect on fasting blood glucose (*F* = 8.8, *df* = 5,330, *P* < .0001) and significant interaction effect on fasting blood glucose (*F* = 3.9, *df* = 25,330, *P* < .0001). The MANOVA results also show significant time period effect on fasting blood glucose (Wilks *λ* = 0.630, *F* = 7.28, *df* = 5,62, *P* < .0001) and significant interaction effect between time periods and groups on fasting blood glucose (Wilks *λ* = 0.332, *F* = 3.20, *df* = 25,231.8, *P* < .0001). 

Tukey's multiple pairwise comparisons test results show that after the induction of T2DM, FBG concentration in T2DM model control group was significantly higher than normal control group (*P* < .01), and at the following 10-week period of drug administration, the FBG in model control group increased with time (*P* < .01); however, there were no significant increases of FBG in T2DM rats treated with encapsulated propolis or positive drug (*P* > .05). No significant differences were found on FBG between model control group and drug-treated groups at the end of the first 4 weeks of drug administration (*P* > .05), while after 6 weeks of drug administration, the FBG concentration in middle- low-dose encapsulated propolis groups and positive control group is significantly lower than model control group (*P* < .01), and after 8 weeks, the FBG concentration in high- middle- and low-dose encapsulated propolis groups and positive control group is significantly decreased than model control group (*P* < .01).

### 3.3. FINS and IAI in T2DM Rats Treated with Encapsulated Propolis

The effects of encapsulated propolis on FINS and IAI in T2DM Rats are given in Table 2. The results show that the level of FINS in T2DM model control group were significantly higher than normal control group (*P* < .01), while in middle-dose encapsulated propolis group and positive control group, the FINS level were decreased significantly than model control group (*P* < .01). The IAI in model control group were significantly lower than normal control group (*P* < .01), treated with encapsulated propolis or positive drug can improve the IAI in T2DM rats, the IAI in high-, middle- and low-dose encapsulated propolis groups and positive control group were significantly higher than model control group (*P* < .01).

### 3.4. TG in T2DM Rats Treated with Encapsulated Propolis

The means and standard deviations of encapsulated propolis on serum TG in T2DM rats are given in Table 3. The results of the time repeated measures ANOVA and MANOVA show significant group effect on TG (*F* = 12.4, *df* = 5,66, *P* < .0001), significant time effect on TG (*F* = 25.7, *df* = 5,330, *P* < .0001) and significant interaction effect on TG (*F* = 6.5, *df* = 25,330, *P* < .0001). The MANOVA results also show significant time effect on TG (Wilks *λ* = 0.409, *F* = 17.9, *df* = 5,62, *P* < .0001) and significant interaction effect between weeks and group on TG (Wilks *λ* = 0.191, *F* = 5.2, *df* = 25,231.8, *P* < .0001).

Tukey's multiple pairwise comparisons test results show that in the remaining 10 weeks of drug administration, the TG concentration in model control group were increased with time (*P* < .01), 4 weeks after drug administration, the TG level in model control group was significantly higher than normal control group(*P* < .01). Encapsulated propolis or positive drug can depress the TG level in T2DM rats; at 4th or 6th week of drug administration, TG level in middle-, low-dose encapsulated propolis groups and positive control group was significantly lower than model control group (*P* < .01, *P* < .05), and after 8 weeks of drug administration, the TG level in all encapsulated propolis treated groups and positive control group were significantly decreased than model control group (*P* < .01).

### 3.5. TC, HDL-C, and LDL-C in T2DM Rats Treated with Encapsulated Propolis

The effects of encapsulated propolis on TC, HDL-C, and LDL-C in T2DM rats are given in Table 4. The results show that there were no significant difference in TC and HDL-C between model control group and normal control group (*P* > .05), while the LDL-C level in model control group were significantly higher than normal control group (*P* < .01). There were no significant difference on TC, HDL-C, and LDL-C level between encapsulated propolis-treated groups and T2DM model groups (*P* > .05), and the LDL-C level in positive control group was significantly lower than model control group (*P* < .05).

### 3.6. Glucose Clamp Study

**Figure 2:**
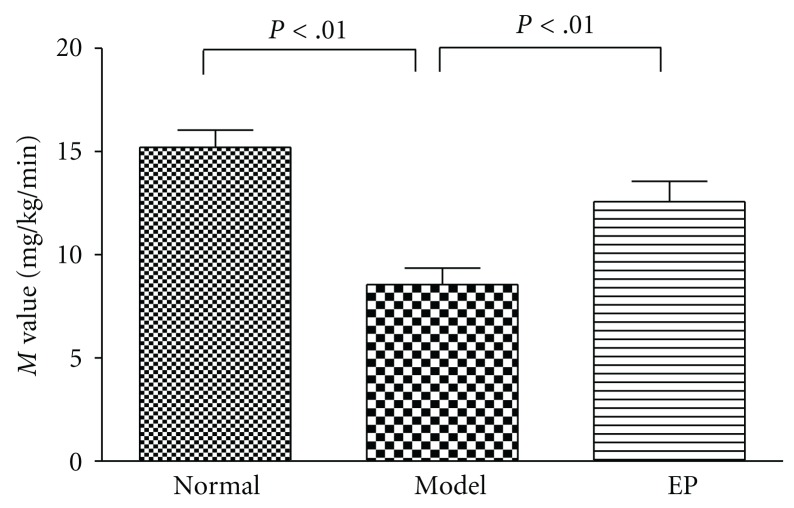
Insulin sensitivity in the normal control and T2DM rats treated with or without encapsulated propolis (100 mg/kg·d) for 6 days. Values are expressed as the means ± standard deviations.

As shown in Figure 2, the average rate of glucose infusion during the last 35 min of glucose clamp, as an index of insulin sensitivity (M value), was significantly lower in the T2DM model control group than normal control group (*P* < .01). Encapsulated propolis (100 mg/kg.d) significantly improved the M value compared to that in model control group (*P* < .01).

## 4. Discussion

Insulin resistance and insulin secrete defection are two important factors in the pathogenesis of T2DM. The results of this study showed that the concentration of FBG and FINS in T2DM model rats was significantly higher than normal rats (*P* < .01), while IAI and the rate of glucose infusion in glucose clamp experiment were significantly lower than normal rats (*P* < .01), indicating the hyperglycemia of this T2DM model we induced was mainly caused by insulin resistance not by insulin secrete defection; the results confirmed those of previous studies that showed feeding healthy rats with a high-fat diet and injected with low-dose STZ results in insulin resistance and hyperglycemia [27].

Unlike type 1 diabetes mellitus, being emaciated is not the feature of T2DM, many T2DM as individuals often suffered obesity. The results showed that the T2DM model rats became middle obesity with the course of T2DM disease prolonging, and the body weight of T2DM model control group were significantly higher than normal control group after 3 weeks handling (*P* < .05); the positive drug pioglitazone HCL tablet increased the body weight of T2DM rats (*P* < .05), which departure the antiobesity treatment policy of T2DM [28]. Otherwise, the results show no significant effect on body weight of T2DM rats treated with encapsulated propolis. 

Hyperglycemia is a macroscopic manifestation of T2DM symptom, and persistence hyperglycemia will affect cells, tissues, and organs all over the body, as it is the main factor to cause diabetes chronic complications. The correlation between risk of microvascular complications and blood glucose control was elucidated by the diabetes control and complications trial (DCCT) and the UK prospective diabetes study (UKPDS), which have provided evidence to link glycemic control and the reduction of risk of diabetic complications [29]. To achieving and maintaining near-normoglycemia is imperative to prevent or delay the onset and progression of long-term complications of the disease. The data obtained from the study showed that encapsulated propolis can depress the heightening of FBG in T2DM rats; 8 weeks after drug administration, the FBG in T2DM rats treated with encapsulated propolis was significantly depressed than T2DM model rats (*P* < .01), indicate encapsulated propolis was efficiency for T2DM blood glucose controlling.

Typically, insulin resistance is an early feature of the T2DM condition, which is initially compensated in part by increased production of insulin by pancreatic *β*-cells (hyperinsulinaemia). Subsequently, as these *β*-cells become “exhausted”, the combined effects of insulin resistance and impaired insulin secretion reduce insulin-mediated glucose uptake and utilization by skeletal muscle and prevent insulin-mediated suppression of hepatic glucose output. Continuing deterioration of endocrine control exacerbates these metabolic disturbances and increases the hyperglycemia [30]. Thus, to improve the insulin sensitivity becomes a very important treatment target in T2DM [31]. In this study, IAI was measured to evaluate the effect of encapsulated propolis on insulin resistance in T2DM rats, and euglycemic hyperinsulinemic clamp, the method considered as “the gold standard” in measuring insulin resistance, was be used to assess accurately the rate of whole-body glucose disposal during steady-state hyperinsulinemia [32]. The results showed the IAI and the average rate of glucose infusion in euglycemic hyperinsulinemic clamp experiment (GIR or M value) were improved significantly than T2DM model rats (*P* < .01), confirming that encapsulated propolis can improve the insulin sensitivity in T2DM rats.

Lipoidosis is a primarily pathological change in T2DM and its long-term complications, as some experts prefered that there might be more rewarding to approach T2DM as “lipocentric” than “glucocentric” [33]. It has long been known that in addition to hyperglycemia, the type 2 diabetic individual almost invariably manifests a serious breakdown in lipid dynamics, often reflected by elevated levels of TG, together with excessive deposition of fat in various tissues including the muscle bed [34, 35]. The depressed activity of insulin-mediated lipoprotein lipase (LPL) made the clearance rate of lipoprotein descend, which is the main cause of hypertriglyceridemia in T2DM disease [36]. The results show that encapsulated propolis can inhabit the increasing of TG levels in T2DM rats, and it is possibly profited by the amendment of insulin sensitivity and LPL activity in treated with encapsulated propolis. The results show no significant difference on TC, LDL-C, and HDL-C between encapsulated propolis treated rats and model rats (*P* > .05). 

It is recommended that appropriate diets, regular exercise, and weight control have potential for the prevention of type 2 diabetes, and ideally, a combination of improved diet, exercise, supplementation, and insulin-sensitizing prescription drugs can reverse mild hyperglycemia to moderate one before stronger drugs are needed. Our data indicated that encapsulated propolis can inhibit the increase of fasting serum glucose, modulate lipid metabolism as well as improve the insulin sensitivity in T2DM rat and, therefore, to delay the development of diabetes. Since this animal model we induced is similar to that of the early-to-moderate stage of T2DM in human beings, the findings provide some evidences of the use of encapsulated propolis in the prevention or in the treatment in the early stages of type 2 diabetes. However, to transfer the results of this animal model to humans requires further investigations.

## 5. Conclusion

Our data indicated that encapsulated propolis exert multiple beneficial effects in fat-fed, streptozotocin-treated type 2 diabetes. Improvement of hyperglycemia and hyperlipidaemia is associated with a marked increase of insulin sensitivity. These findings further support the use of encapsulated propolis in the treatment of type 2 diabetes and emphasize the possibility that the deterioration of blood glucose concentration over time may be prevented.

## Figures and Tables

**Table 1:** Effects of encapsulated propolis on FBG in T2DM rats ($\overset{̅}{x}$  ± S.D.).

Group	Dosage	*n*	Fasting blood glucose (mmol/L)					
	(administration)		0 week	2 week	4 week	6 week	8 week	10 week
Normal control	1 mL/100 g distilled water (*p.o*.)	12	6.07 ± 0.31**	6.18 ± 0.21**	6.09 ± 0.28**	6.09 ± 0.24**	6.13 ± 0.24**	6.19 ± 0.33**

Model control	1 mL/100 g distilled water (*p.o*.)	12	7.35 ± 0.69	7.39 ± 0.46	7.51 ± 0.76	8.23 ± 0.84	8.61 ± 0.73	9.05 ± 1.06

Low-dose EP	50 mg/kg encapsulated propolis (*p.o*.)	12	7.30 ± 0.77	7.82 ± 0.52	7.67 ± 0.69	7.13 ± 0.52**	7.74 ± 0.49**	7.88 ± 0.46**

Middle-dose EP	100 mg/kg encapsulated propolis (*p.o*.)	12	7.20 ± 1.73	7.79 ± 0.70	7.68 ± 0.64	7.51 ± 0.50**	7.59 ± 0.40**	7.51 ± 0.50**

High-dose EP	200 mg/kg encapsulated propolis (*p.o*.)	12	7.17 ± 1.20	7.68 ± 0.84	7.63 ± 0.85	7.67 ± 0.41	7.32 ± 0.60**	7.37 ± 0.68**

Positive control	10 mg/kg Pioglitazone HCL (*p.o*.)	12	7.28 ± 1.45	7.68 ± 0.72	6.93 ± 0.64	7.01 ± 0.47**	7.53 ± 0.45**	7.54 ± 0.46**

Tukey post-hoc tests: *P < .05 and **P < .01 compared with model control group.

**Table 2:** Effects of encapsulated propolis on FINS and IAI in T2DM Rats ($\overset{̅}{x}$  ± S.D.).

Group	Dosage (administration)	*n*	Fasting serum insulin (*μ*IU/mL)	Insulin action index
Normal control	1 mL/100 g distilled water (*p.o*.)	12	39.75 ± 10.85**	−5.44 ± 0.30**

Model control	1 mL/100 g distilled water (*p.o*.)	12	55.85 ± 7.87	−6.17 ± 0.22

Low-dose EP	50 mg/kg encapsulated propolis (*p.o*.)	12	43.65 ± 8.32	−5.79 ± 0.27**

Middle dose EP	100 mg/kg encapsulated propolis (*p.o*.)	12	40.80 ± 10.99**	−5.60 ± 0.55**

High-dose EP	200 mg/kg encapsulated propolis (*p.o*.)	12	44.21 ± 5.50	−5.76 ± 0.14**

Positive control	10 mg/kg Pioglitazone HCL (*p.o*.)	12	40.55 ± 14.88**	−5.64 ± 0.20**

The data were measured at the end of study. *P < .05 and **P < .01 compared with model control group.

**Table 3:** Effects of encapsulated propolis on TG in T2DM rats ($\overset{̅}{x}$  ± S.D.).

Group	Dosage	*n*	Serum triglycerides (mmol/L)					
	(administration)		0 week	2 week	4 week	6 week	8 week	10 week
Normal control	1 mL/100 g distilled water (*p.o*.)	12	1.67 ± 0.40	1.89 ± 0.34	1.78 ± 0.38**	1.80 ± 0.27**	1.82 ± 0.39**	2.17 ± 0.37**

Model control	1 mLl/100 g distilled water (*p.o*.)	12	1.56 ± 0.34	1.59 ± 0.34	2.89 ± 0.70	2.98 ± 0.58	3.09 ± 0.54	3.08 ± 0.74

Low-dose EP	50 mg/kg encapsulated propolis (*p.o*.)	12	1.60 ± 0.42	1.77 ± 0.55	2.27 ± 0.53*	2.10 ± 0.38**	2.15 ± 0.60**	2.32 ± 0.59

Middle-dose EP	100 mg/kg encapsulated propolis (*p.o*.)	12	1.65 ± 0.37	1.79 ± 0.51	2.00 ± 0.26**	1.78 ± 0.46**	1.50 ± 0.45**	1.54 ± 0.59**

High-dose EP	200 mg/kg encapsulated propolis (*p.o*.)	12	1.68 ± 0.44	1.84 ± 0.52	2.43 ± 0.49	2.48 ± 0.47	2.18 ± 0.58**	2.13 ± 0.65**

Positive control	10 mg/kg Pioglitazone HCL (*p.o*.)	12	1.75 ± 0.55	1.60 ± 0.36	2.13 ± 0.47**	1.87 ± 0.76**	1.98 ± 0.69**	1.59 ± 0.46**

Tukey post hoc tests: *P < .05 and **P < .01 compared with model control group.

**Table 4:** Effects of encapsulated propolis on TC, HDL-C, LDL-C in T2DM Rats ($\overset{̅}{x}$  ± S.D.).

Group	Dosage (administration)	*n*	Total cholesterol	HDL cholesterol (mmol/L)	LDL cholesterol
Normal control	1 mL/100 g distilled water (*p.o*.)	12	2.10 ± 0.47	1.02 ± 0.19	0.21 ± 0.08**

Model control	1 mL/100 g distilled water (*p.o*.)	12	2.24 ± 0.28	1.09 ± 0.26	0.32 ± 0.07

Low-dose EP	50 mg/kg encapsulated propolis (*p.o*.)	12	2.22 ± 0.44	1.14 ± 0.21	0.27 ± 0.05

Middle-dose EP	100 mg/kg encapsulated propolis (*p.o*.)	12	2.34 ± 0.48	1.12 ± 0.19	0.29 ± 0.06

High-dose EP	200 mg/kg encapsulated propolis (*p.o*.)	12	2.15 ± 0.35	1.11 ± 0.17	0.26 ± 0.05

Positive control	10 mg/kg Pioglitazone HCL (*p.o*.)	12	2.00 ± 0.50	0.90 ± 0.18	0.24 ± 0.02*

The data were measured at the end of study. *P < .05 and **P < .01 compared with model control group.
